# Feasibility, criterion and construct convergent validity of the 2-minute walk test and the 10-meter walk test in an oncological context

**DOI:** 10.1016/j.heliyon.2023.e22180

**Published:** 2023-11-10

**Authors:** Aline Reinmann, Thibaud Koessler, Alexandre Bodmer, Axelle Baud-Grasset, Géraldine Mentha, Joseph Gligorov, Anne-Violette Bruyneel

**Affiliations:** a Geneva School of Health Sciences, HES-SO University of Applied Sciences and Arts Western Switzerland, Geneva, Switzerland; b Sorbonne University, INSERM U938, Centre de Recherche Saint Antoine, CRSA, Paris, France; c Service of Oncology, Geneva University Hospitals, Geneva, Switzerland; d University of Geneva, Geneva, Switzerland; e University Institute of Cancerology AP-HP Sorbonne University, Medical Oncology site Tenon, Paris, France

**Keywords:** Cancer, Validity, Walking, 6-Minute walk test, 2-Minute walk test, 10-Meter walk test

## Abstract

**Objective:**

To examine the feasibility, the criterion, and the construct convergent validity of the 2-Minute Walk Test (2MWT) and the 10-Meter Walk Test (10MeWT) against the 6-Minute Walk Test (6MWT) to assess walking capacity in people with cancer. The criterion concurrent validity of a self-test version of the 10MeWT (10MeWT_self-test_) was also evaluated against the 10MeWT.

**Methods:**

Fifty-six people with cancer performed the 2MWT, the 10MeWT at comfortable and fast speeds, the 6MWT, and the 10MeWT_self-test_. The feasibility of the tests was assessed using safety, adverse events, space requirements, time taken to administer and interpret the tool, equipment or training required, cost, and portability as criteria. Validity was assessed using Pearson correlation coefficients and Bland Altman plots.

**Results:**

The 2MWT, 6MWT, 10MeWT, and 10MeWT_self-test_ were feasible for people with cancer. The 2MWT and the 10MeWT results were moderately to strongly correlated with the 6MWT results (0.61 < *r* < 0.84, *p* < 0.001). The 10MeWT_self-test_ results were strongly correlated with the 10MeWT results at comfortable and fast speeds (*r* = 0.99, *p* < 0.001).

**Conclusions:**

The 2MWT, 10MeWT, and 10MeWT_self-test_ are simple, rapid, and feasible tests for use in people with cancer. The strong correlation between the 2MWT and 6MWT results indicates that the 2MWT can be used as an alternative walking capacity assessment tool. The 10MeWT results moderately correlated with those of the other two tests, suggesting that it partially measures the same construct of walking capacity in walking-independent outpatients with cancer. The 10MeWT_self-test_ showed promising results but needs further investigations in ecological settings.

## 1 Introduction

More than one in two people with cancer have walking difficulties [1]. The pathology [2], the toxicity of treatments [3,4] and the lack of physical activity, which affects up to 90 % of patients during treatment [2,5], can have numerous repercussions on both the cardio-respiratory and neuro-muscular systems. Decreases in peak oxygen consumption and altered cardiac function have been reported and could contribute to reduced walking capacity [2,3], defined as ‘what the person can do’ in terms of walking [6]. Similarly, loss of muscle mass and function [7] could negatively impact walking capacity, as could alterations in the central (neuromuscular fatigue) [8] and peripheral nervous systems (sensorimotor alterations) [4]. Furthermore, such impairments could also reduce postural control and dynamic balance [1,9]. Impaired walking capacity associated with activity limiting symptoms, like pain, fatigue and depression, can lead to functional disability and have serious repercussions on quality of life [1]. Rehabilitation can be set up to prevent functional decline or improve walking capacity [10]; however, as for all treatments, it is essential to measure baseline values and to set targets that can be re-evaluated at follow-up visits [11]. It is important to use objective tests that are easy to perform to screen for difficulties, potentially even before patients start to feel limitations in their daily activities [12].

The tests should have good to excellent validity and reliability and be feasible for use in the clinical setting. Considerations include safety, adverse events, space required, test duration, equipment required, training, cost, portability of test equipment and the overall burden of performing the test for both clinicians and patients [11,13,14]. One of the most widely used field tests to quantify walking capacity is the 6-Minute Walk Test (6MWT) [15]. This test consists of walking as far as possible at a self-selected pace for six minutes [16]. This test, has excellent test-retest reliability in individuals with cancer [11], is safe, inexpensive, and requires little equipment. However, although the 6MWT is widely used and recommended by the Oncology Edge Task Force [13,17,18], it has limitations. In addition to requiring a large space (30 meters walking distance), the 6MWT may be too difficult for people with cancer to perform because of their illness or the side effects of treatment [19]. Moreover, the 6MWT may expose people to fall risks caused by balance disorders, which are highly prevalent in this population [20]. Its relatively long duration – about 15 minutes including preparation and recovery time [21] – reduces the time available for testing other motor capacities impacted by the disease. Thus, a shorter test might improve the feasibility of use in the clinical setting and decrease the evaluation duration for individuals with cancer.

The shortened version of the 6MWT, the 2-Minute Walk Test (2MWT), which follows the same procedure as the 6MWT [22], may be less burdensome and time consuming for both individuals with cancer and clinicians [23]. Excellent construct validity with the 6MWT and excellent test-retest, inter-reliability, and intra-reliability of the 2MWT were found in community-dwelling adults [[24], [25], [26]], older adults living in long-term care [22,27], and people with neurological [23,28] and pulmonary diseases [29]. This test is increasingly used in oncology to assess the effect of an intervention [30,31] or to evaluate changes in function after an intervention [32]. Two guidelines from the Oncology EDGE Task Force have recommended its use to assess walking capacity in people with breast and prostate cancer because of its good psychometric properties, ease of administration, and the availability of normative data [13,17]. Despite this, no previous studies have evaluated the validity of the 2MWT for use with individuals with cancer.

The 10-Meter Walk Test (10MeWT) is also an attractive option to easily determine the presence of physical impairments that limit the person's activities and participation [17]. By measuring comfortable and fast walking speed, this test quickly provides information on the person's functional capabilities and safety according to pre-established thresholds [33]. The ease of test administration, the information provided, and the excellent test-retest reliability in people with cancer make the 10MeWT relevant for clinical practice [11]. Moreover, given its simplicity, the 10MeWT could be performed by the individual in a self-testing version [34]. This could be an interesting variant for use in telerehabilitation, which has recently gained prominence [35].

It is not yet established in the literature to what extent the 10MeWT measures the same construct of walking capacity as the 2MWT and 6MWT in an oncological context. Although the two longer tests are known to assess the endurance and exercise tolerance aspects of walking capacity, the 10MeWT is a rapid test of general functional capacity [11]. Despite this difference, more than a dozen studies conducted in people with mainly neurological disorders have reported good to strong correlations between the 10MeWT and the 6MWT or the 2MWT (Supplementary material). No studies have been conducted in samples of people with different cancers, despite the fact that cancer has specific physical repercussions affecting walking capacity. In oncology, only Eden et al. (2018) evaluated people with head and neck cancer and found a moderate correlation between the 10MeWT and the 6MWT [11]. That study was based on a specific population and did not include the 2MWT. A study integrating people with various types of cancer and including different tests would improve the evidence base for walking tests in oncology and facilitate clinical decisions such as determining rehabilitation needs or planning specific care.

The main objective of this study was to assess the feasibility of the 2MWT, the 6MWT and the 10MeWT in people with cancer. The secondary objective was to evaluate the validity of these tests to assess walking capacity. Other aims were to perform exploratory analyses by age subgroups, as age has a significant influence on comfortable and fast walking capacities [36], and to determine the feasibility of a self-tested version of the 10MeWT (10MeWT_self-test_) and its validity compared to the therapist-evaluated 10MeWT. We hypothesized that the 2MWT, the 6MWT and the 10MeWT would be feasible for use with people with cancer and that the results of each test would be moderately to strongly correlated. In addition, we hypothesized that the 10MeWT_self-test_ would be feasible for use with people with cancer, given its ease of use, and that the results of the 10MeWT_self-test_ would be strongly correlated with the results of the therapist-evaluated 10MeWT.

## 2 Materials and methods

### 2.1 Participants

According to the Consensus-based Standards for the selection of health Measurement Instruments (COSMIN) recommendations, a minimum of 50 participants are required to assess the criterion and construct validity of a test [37]. To participate in the study, people with an oncological disease had to be able to walk without technical aids for six minutes, be over 18 years old, and have medical authorization to perform the test. Individuals were excluded if they had contraindications to taking part in walking fields tests (unstable heart problems, or inability to understand the instructions) [16], if they had severe treatment side-effects, like severe fatigue, that prevented following the test procedure, or if they had pain scores >2/10 during walking that could have influenced the test results and caused discomfort to the participant. Participants were all outpatients, prospectively recruited at the University Hospital of Geneva during a follow-up appointment with the oncologist. After being screened for eligibility, they were given information about the study by a person not involved in their care, including the purpose of the study, procedures, benefits, and risks. The individual then had to give written consent to participate in the study after receiving an information letter and being provided with the opportunity to ask questions.

The inclusion rate, corresponding to the rate of people who agreed to participate in the study among all the people informed about the study was calculated.

### 2.2 Design and study procedure

We conducted an observational, cross-sectional validity study. Four walking tests (6MWT, 2MWT, 10MeWT, 10MeWT_self-test_) were administered by the same rater in a randomized order. The order of testing was defined by randomly drawing tests from an opaque envelope during the pre-test rest period. Between tests, participants rested in a sitting position for a duration adapted to their heart rate (HR, bpm) and saturation (SpO_2_, %) values and the strenuousness of the effort rated on a BORG 0–10 scale [38]. The rest period was stopped when the values were similar to the pre-test HR and SpO_2_ values.

A single leg stance test was added to characterize the population. The tests were all carried out between 11 a.m. and 4 p.m. in the rehabilitation department of the University Hospital of Geneva. The floor was hard, flat, and non-slip. The tests were conducted by three physiotherapists familiar with field walking tests. Test standardization sessions and an audit were established to ensure that all raters used the same procedures and that the general protocol was respected.

### 2.3 Tests

The 6MWT was conducted in a 30-meter corridor graded every 5 meters according to the guidelines of the European Respiratory Society/American Thoracic Society [16]. Participants were asked to walk “as far as possible for six minutes by walking back and forth along the corridor” [39] and were encouraged using standardized incentives [39]. The distance walked was measured at two and six minutes. HR, SpO_2_, and strenuousness of the effort were recorded before, and at two, four, and six minutes. Fatigue was recorded before and at six minutes. HR and SpO_2_ were measured by a fingertip pulse oximeter (Onyx® Vantage 9590, 2020, Nonin, Minneapolis, USA), considered a reliable technique for the detection of a noteworthy decline in respiratory function with a concern threshold set at 80 % saturation [40,41]. The strenuousness of the effort and fatigue were collected by asking the participants to rate their feeling from 0 (no strenuousness or fatigue at all) to 10 (maximal strenuousness or exertion) [42]. The 6MWT was discontinued if the participant had pain >2/10 on the numerical rating scale (NRS), desaturation <80 % SpO_2_, or a significant fall risk. The 6MWT has good psychometric properties, including excellent test-retest reliability (Intraclass Correlation Coefficient, *ICC* = 0.96) in people with cancer [11]. The minimum detectable change (MDC) is established at 28.1 meters and the standard error of measurement (SEM) at 10.1 meters in older adults with dementia [43].

The 2MWT was conducted according to the 6MWT procedure [16]. HR, SpO_2_, strenuousness, and fatigue were recorded before and at two minutes. Good to excellent test-retest (*ICCs* range: 0.82–1), inter-reliability (*ICCs* range: 0.85–0.96) and intra-reliability (*ICC* = 0.85) of the 2MWT were found for this test in community-dwelling adults [24,25], older adults living in long-term care [22,43], and in people with neurological [28] and pulmonary diseases [29]. The MDC and SEM are respectively 15 meters and 6.3 meters in older adults [22].

The 10MeWT involves measuring the time needed to cover a 10-meter distance at a comfortable and fast speed with a stopwatch in a hospital corridor. Walking speed is then calculated. The measurement using a stopwatch has excellent concurrent validity for measuring walking speed [44]. The test was performed over 14 meters to allow two meters of acceleration and deceleration [33], and was repeated three times [11]. Using a short video explaining how to perform the test, participants were asked to “walk at their usual speed, like when going to the shops, as far as the white line” and then to “walk as fast as possible to the white line”. The participants and the rater simultaneously measured the time to walk 10 meters. The measurement performed by the participant and the explanatory video were intended to assess the feasibility of performing the test independently at home. The reliability of the 10MeWT is excellent in people with cancer (*ICC* = 0.94) [11], and the MDC and SEM are respectively 0.16 meters/second, and 0.06 meters/second in older adults with dementia [43].

The single leg balance stance test was performed with eyes open, on the leg chosen by the participants, with subjects holding their arms at their sides [45]. The test was interrupted if the duration exceeded 60 seconds. The best duration of the two trials carried out was selected [45].

#### 2.3.1 Statistical analyses

Analyses were conducted with Stata (v.15, 2017, Stata Corporation, USA). First, the mean of the three trials of the 10MeWT was calculated, and the walking speed of the three tests was calculated using the following formula: distance (*m*)/time (*s*) = walking speed (*m/s*). Percentages were calculated for categorical variables and means ± standard deviations (SD) were calculated for continuous variables.

The feasibility of the 2MWT, 10MeWT, and 10MeWT_self-test_ was determined according to test safety (desaturation <80 % SpO2), adverse events (pain affecting walking >2/10 on NRS, fall), space required, as well as on Tyson and Connell items (2009): the time taken to administer and interpret the tool, the equipment or training required, the cost, and the portability of the measurement tool [14]. A score of 9/10 was required to recommend the tool for use in clinical practice.

After checking the normality of the data distribution, Pearson correlations were performed to assess the criterion validity of the 2MWT against the 6MWT, and the convergent construct validity of the 10MeWT against the 6MWT and the 2MWT. A Bland-Altman plot was used to visualize the agreement between the 2MWT and the first two minutes of the 6MWT. The limits of agreement (LOA 95 %) were calculated (mean difference between the two tests ± 1.96 * *SD* of the difference between the tests). Given the age, heterogeneity of people with cancer and the effect of age on walking capacity [36], exploratory analyses by age (<65 and ≥65 years) were also conducted. Correlations by age subgroups were performed. The concurrent criterion validity of the 10MeWT_self-test_ against the 10MeWT was assessed using Pearson correlations and a Bland-Altman plot. A correlation of 0.90 was considered very strong, 0.70–0.89 strong, 0.40–0.69 moderate, 0.10–0.39 weak, and less than 0.1 negligible [46]. For all analyses, the *p*-value for two-tailed significance level was set at *p* < 0.05.

## 3 Results

### 3.1 Demographic characteristics

All participants were recruited and tested between February and March 2021. The inclusion rate was 25.38 %. The main reason for refusal was too many appointments already scheduled. The other reasons were not being able to perform the tests without walking aids, not wishing to take part in the study, having pain >2/10 that could impact walking, not understanding French, being too tired to participate, and having cognitive impairments. All participants completed the study procedure except one who experienced leg pain greater than 2/10 during the 6MWT. Thus, data from 56 participants were analyzed for all tests, except for the 6MWT (*n* = 55). Participants were 65.5 ± 11.9 years of age, and the most common type of cancer was lymphoma/leukemia followed by lung and colon cancer. Most participants were treated with chemotherapy (Table 1).

**Table 1:** Baseline participant characteristics.

Characteristics	Values
**Entire sample**	
Age (years)	65.5 ± 11.9 (36–90)
Height (cm)	170.6 ± 8.9 (150–192)
Weight (kg)	71.3 ± 14.3 kg (41.5–118)
Body Mass Index (kg/m^2^)	24.5 ± 4.4 (14.7–42.3)
Women (n, %)	17 (30)
**Time since diagnosis (years)**	3.7 ± 4.1 (0–16)
**Cancer type (n, %)**	
Lymphoma/Leukemia	12 (21)
Lung	9 (16)
Colon	5 (9)
Rectal	3 (5)
Breast	3 (5)
Ovarian	3 (5)
Prostate	2 (4)
Liver	2 (4)
Pancreatic	2 (4)
Esophageal	2 (4)
Oropharyngeal	2 (4)
Bladder	2 (4)
Brain	2 (4)
Myeloma	1 (2)
Skin	1 (2)
Sarcoma	1 (2)
Renal	1 (2)
Small bowel	1 (2)
Erdheim Chester	1 (2)
Gall bladder	1 (2)
Carcinoma of unknown origin	1 (2)
**Metastasis (n, %)**	
Yes	25 (45)
No	31 (55)
**Treatment intent (n, %)**	
Curative	30 (55)
Palliative	16 (29)
Other (in remission)	9 (16)
**Current therapy (n, %)**	
Chemotherapy	14 (25)
Immune therapy	9 (16)
Targeted therapy	5 (9)
Hormone therapy	3 (5)
Chemotherapy + Radiation therapy	2 (4)
Chemotherapy + Targeted therapy	2 (4)
Chemotherapy + Immune therapy	2 (4)
Hormone therapy + Radiation therapy	1 (2)
Immune therapy + Targeted therapy	1 (2)
None	17 (30)
**Balance capacity**	
Single Leg Stance Test (sec)	32.6 ± 22.2 (0.7–60)
**Sub-group <65 years old**	
Age (years)	53.9 ± 6.5 (36–63)
Height (cm)	172 ± 9.2 (158–185)
Weight (kg)	72.7 ± 16.4 (45–118)
Body Mass Index (kg/m^2^)	24.6 ± 5.5 (17.8–42.3)
Women (n, %)	3 (13)
**Sub-group ≥65 years old**	
Age (years)	73.7 ± 6.9 (65–90)
Height (cm)	169.6 ± 8.7 (150–192)
Weight (kg)	70.4 ± 12.9 (41.5–92)
Body Mass Index (kg/m^2^)	24.4 ± 3.5 (14.7–29.8)
Women (n, %)	14 (42)

Table 1. Cancer-related characteristics of included participants. Data are mean ± SD (min – max) or n (%). N = 56 except for the period of illness (n = 55), one missing data for the <65 years sub-group (n = 23) and for the ≥65 years sub-group (n = 33).

### 3.2 Walking tests results

Mean walking speeds and distances of the different tests are shown in Table 2.

**Table 2:** 6MWT, 2MWT and 10MeWT values.

Variables	2MWT	6MWT	10MeWT
**Entire sample**			
Speed (m/s)	1.34 ± 0.31 (0.76–2.38)	1.31 ± 0.33 (0.70–2.17)	1.26 ± 0.26 (0.74–1.93)
Speed first 2 min (m/s)	–	1.31 ± 0.30 (0.71–2.12)	–
Speed last 4 min (m/s)	–	1.30 ± 0.36 (0.59–2.19)	–
Fast speed (m/s)	–	–	1.67 ± 0.36 (0.92–2.46)
Self-test comfortable speed (m/s)	–	–	1.27 ± 0.27 (0.73–1.93)
Self-test fast speed (m/s)	–	–	1.69 ± 0.37 (0.96–2.49)
Distance 2 min (m)	160.63 ± 37.34 (91–285)	157.47 ± 36.14 (85–254)	–
Distance 6 min (m)	–	469.89 ± 117.07 (252–780)	–
BORG (/10)			
Pre	1.77 ± 1.57 (0–6)	1.33 ± 1.49 (0–5)	–
Two minutes	4.11 ± 1.77 (1-8)	3.58 ± 1.62 (0–6)	–
Four minutes	–	4.04 ± 1.67 (0–8)	–
Six minutes	–	4.55 ± 1.64 (1-8)	–
SpO_2_ (%)			
Pre	97.73 ± 0.94 (95–100)	97.82 ± 1.04 (96–100)	–
Two minutes	95.98 ± 2.73 (84–100)	96.06 ± 2.15 (90–99)	–
Four minutes	–	95.87 ± 2.97 (84–100)	–
Six minutes	–	95.58 ± 3.60 (80–99)	–
HR (bpm)			
Pre	80.20 ± 14.94 (61–141)	79.04 ± 14.00 (56–144)	–
Two minutes	101.95 ± 16.57 (45–136)	103.29 ± 15.56 (65–145)	–
Four minutes	–	105.04 ± 16.50 (64–149)	–
Six minutes	–	107.44 ± 15.25 (79–159)	–
Fatigue (/10)			
Pre	2.96 ± 2.09 (0–8)	2.80 ± 2.10 (0–8)	–
Post	3.63 ± 2.17 (0–8)	3.76 ± 2.12 (0–8)	–
Recuperation SpO_2_, (n, %)			
<60 s	30 (54)	33 (60)	–
60–180 s	23 (41)	17 (31)	–
>180 s	3 (5)	5 (9)	–
Recuperation HR (sec)			
<60 s	15 (27)	6 (11)	–
60–180 s	31 (55)	28 (52)	–
>180 s	10 (18)	20 (37)	–
Break			
N participant with break (n, %)	0	2 (4)	–
Break duration (sec)	0	47.33 ± 36.46 (8–80)	–
**Sub-group < 65 years**			
Speed (m/s)	1.41 ± 0.29 (0.96–2.04)	1.47 ± 0.32 (0.90–2.17)	1.35 ± 0.27 (0.90–1.93)
Fast speed (m/s)	–	–	1.85 ± 0.34 (1.31–2.46)
**Sub-group ≥ 65 years**			
Speed (m/s)	1.29 ± 0.32 (0.76–2.38)	1.19 ± 0.28 (0.70–1.76)	1.20 ± 0.24 (0.74–1.61)
Fast speed (m/s)	–	–	1.55 ± 0.33 (0.92–2.11)

Table 2. 2MWT, 6MWT and 10MeWT values. Data are mean ± SD (min – max). HR = Heart Rate, SpO_2_ = saturation, 6MWT = 6-Minute Walk Test, 2MWT = 2-Minute Walk Test, 10MeWT = 10-Meter Walk Test. (n = 56) except for 6MWT values (n = 55), and for fatigue and recuperation HR of 6MWT two missing values (n = 54).

### 3.3 Feasibility

No safety issues were noted, however, one participant had to stop during the 6MWT because of leg pain >2/10 and two had to take a break during the last four minutes of the test because of the intensity of the effort. All participants were able to perform the 10MeWT and 2MWT without safety concerns or adverse events. According to Tyson and Connell's (2009) scale, the 2MWT, the 6MWT, and the 10MeWT were feasible (score ≥9/10) [14] (see Table 3 for details) but the 10MeWT required less space (14 meters versus 34 meters). The self-test version of the 10MeWT was also feasible, except for the video instructions that needed to be re-explained by the examiners for almost all the participants.

**Table 3:** Feasibility of the tests.

	2MWT	6MWT	10MeWT & 10MeWT_self-test_
**Time taken to administer, analyze and interpret**	3	2	3
**Cost**	3	3	3
**Specialist equipment and training**	2	2	2
**Measurement tool portable**	2	2	2
**Total**	10	9	10

Table 3. Scores for the feasibility of the tests according to Tyson and Connell items (2009). 6MWT = 6-Minute Walk Test, 2MWT = 2-Minute Walk Test, 10MeWT = 10-Meter Walk Test.

### 3.4 Validity

**Fig. 1:**
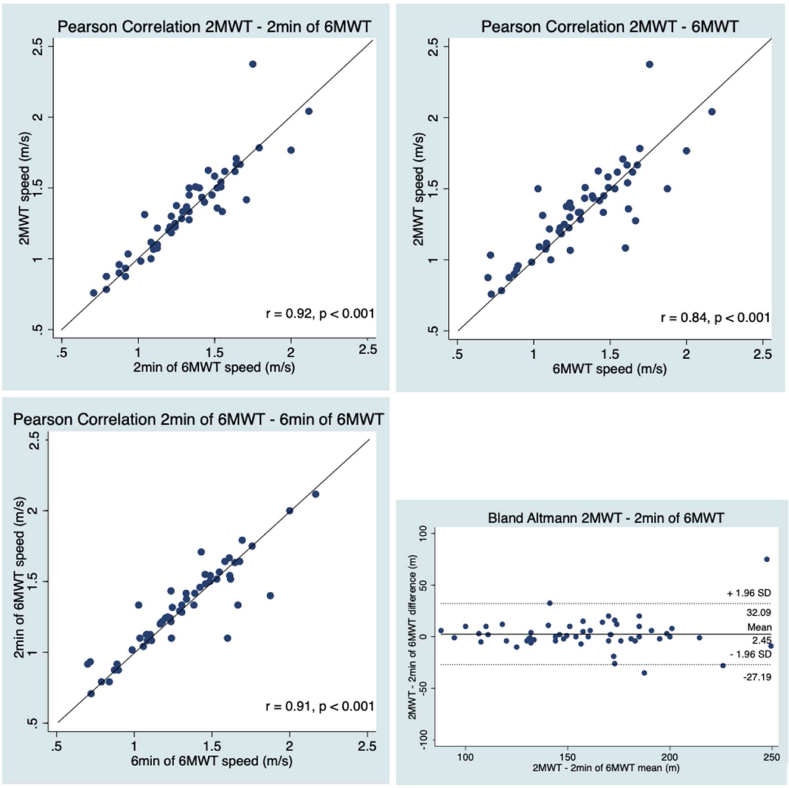
Pearson correlations between the 2MWT and the first two minutes of the 6MWT, the 2MWT and the 6MWT, the first two minutes of the 2MWT and the 6MWT, and Bland Altman of the 2MWT and the first two minutes of the 6MWT. Solid line = perfect correlation line for scatter plots and mean difference for the Bland Altmann plot. Dotted line = mean difference ± 1.96 SD. P = p-value, r = correlation, SD = Standard Deviation, 2MWT = 2-Minute Walk Test, 6MWT = 6-Minute Walk Test. (n = 55).

Concerning the criterion validity of the 2MWT against the 6MWT, the 2MWT results were strongly to very strongly correlated with the 6MWT results for the whole group and both subgroups (0.82 ≤ *r* ≥ 0.94, *p* < 0.001, Table 4 and Table 5, Fig. 1). The Bland-Altman plot showed no systematic difference between the results from the 2MWT and the two minutes of the 6MWT and an even distribution of differences across the range of values (Fig. 1). The mean difference was 2.50 ± 15.1 meters with an upper 95 % LOA of 32.10 meters and a lower 95 % LOA of −27.10 meters.

**Table 4:** Pearson coefficient correlation.

	2MWT	Two minutes 6MWT	6MWT	10MeWT comfortable speed	10MeWT fast speed	10MeWT _self-test_
**2MWT**	–	0.92	0.84	0.64	0.68	–
**Two minutes 6MWT**	0.92	–	0.91	0.69	0.74	–
**6MWT**	0.84	0.91	–	0.61	0.71	–
**10MeWT comfortable speed**	0.64	0.69	0.61	–	–	0.99
**10MeWT fast speed**	0.68	0.74	0.71	–	–	0.99
**10MeWT**_**self-test**_	–	–	–	0.99	0.99	–

Table 4. Pearson correlation coefficients. P-value <0.001 for all analyses. 6MWT = 6-Minute Walk Test, 2MWT = 2-Minute Walk Test, 10MeWT = 10-Meter Walk Test. (n = 56) except for 6MWT values (n = 55).

**Fig. 2:**
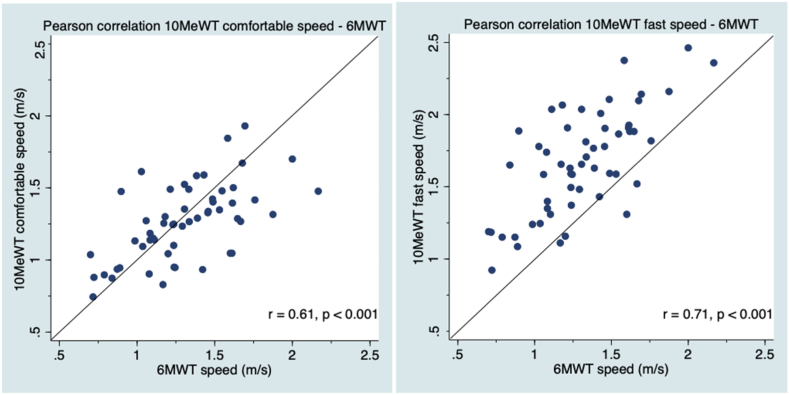
Pearson correlations between the 10MeWT and the 6MWT at comfortable and fast speeds. Solid line = perfect correlation line. P = p-value, r = correlation, 10MeWT = 10-Meter Walk Test, 6MWT = 6-Minute Walk Test. (n = 55).

Concerning the construct validity of the 10MeWT against the 6MWT, the 10MeWT results were moderately to strongly correlated with the 6MWT results for comfortable and fast speeds (0.61 ≤ *r* ≥ 0.71, *p* < 0.001, Table 4, Table 5, Fig. 2). The correlation coefficients between these tests were higher in the older individuals than in the younger individuals at comfortable (*r* = 0.65, *p* < 0.001 vs *r* = 0.47, *p =* 0.029) and fast speeds (*r* = 0.71, *p* < 0.001 vs *r* = 0.56, *p* = 0.007).

**Table 5:** Pearson coefficient correlation for each age subgroup.

Sub-group <65 years						
	2MWT	Two minutes 6MWT	6MWT	10MeWT comfortable speed	10MeWT fast speed	10MeWT _self-test_
**2MWT**	–	0.94	0.82	*0.58*	0.66	–
**Two minutes 6MWT**	0.94	–	–	–	–	–
**6MWT**	0.82	–	–	*0.47*	*0.56*	–
**10MeWT comfortable speed**	*0.58*	–	*0.47*	–	–	1.00
**10MeWT fast speed**	0.66	–	*0.56*	–	–	0.99
**10MeWT**_**self-test**_	–	–	–	1.00	0.99	–
**Sub-group ≥65 years**						
	**2MWT**	**Two minutes 6MWT**	**6MWT**	**10MeWT comfortable speed**	**10MeWT fast speed**	**10MeWT**_**self-test**_
**2MWT**	–	0.93	0.90	0.66	0.68	–
**Two minutes 6MWT**	0.93	–	–	–	–	–
**6MWT**	0.90	–	–	0.65	0.71	–
**10MeWT comfortable speed**	0.66	–	0.65	–	–	0.99
**10MeWT fast speed**	0.68	–	0.71	–	–	0.99
**10MeWT**_**self-test**_	–	–	–	0.99	0.99	–

Table 5. Pearson correlation coefficients for age subgroups. P-value <0.001 for all analyses except those in italics where p-value <0.05. 6MWT = 6-Minute Walk Test, 2MWT = 2-Minute Walk Test, 10MeWT = 10-Meter Walk Test. (n = 56) except for 6MWT values (n = 55).

Concerning the construct validity of the 10MeWT with the 2MWT, moderate correlations were found between the 10MeWT and the 2MWT results for comfortable and fast walking, in the whole group and the subgroups (0.58 ≤ *r* ≥ 0.68, *p* < 0.010, Table 4, Table 5).

**Fig. 3:**
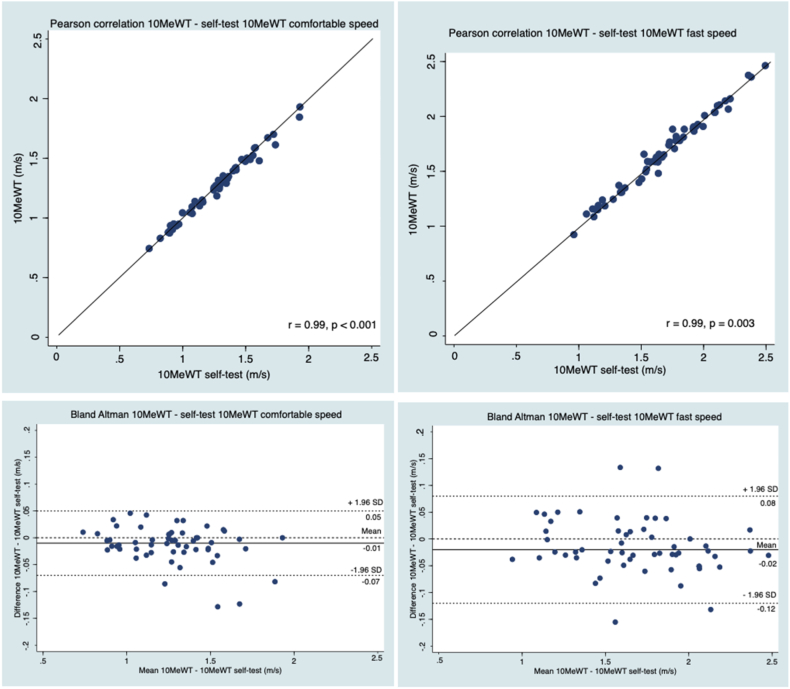
Pearson correlations between the 10MeWT and the 10MeWT_self-test_ at comfortable and fast speeds, and Bland Altman of the 10MeWT and the 10MeWT_self-test_. Solid line = perfect correlation line for scatter plots and mean difference for the Bland Altmann plot. Dotted line = mean difference ± 1.96 SD. P = p-value, r = correlation, SD = Standard Deviation, 10MeWT = 10-Meter Walk Test. (n = 55).

Concerning the concurrent criterion validity between the 10MeWT_self-test_ and the 10MeWT, the correlation coefficients were strong for the whole group and the age subgroups at both comfortable and fast speeds (0.99 ≤ *r* ≥ 1.00, *p* < 0.001, Table 4 and 5, Fig. 3). The Bland-Altman plots showed no systematic difference between the tests (Fig. 3). The mean difference was −0.01 ± 0.03 meters for comfortable walking with an upper 95 % LOA of 0.05 meters and a lower 95 % LOA of −0.07 meters; and −0.02 ± 0.05 meters for the fast speed with an upper LOA 95 % of 0.08 meters and a lower LOA 95 % of −0.12 meters.

## 4 Discussion

Performance of the 2MWT, 6MWT and 10MeWT was feasible in the sample of people with cancer. The results of all three tests were moderately to very strongly correlated with each other. Moreover, the concurrent validity of the self-test version of the 10MeWT was excellent against the therapist-evaluated version. Compared to the 6MWT, the 2MWT and the 10MeWT allow clinicians to easily test walking capacity in people with cancer with fewer constraints.

In addition to being less time consuming, the 2MWT and 10MeWT were easily completed by all participants. Shorter tests seem advantageous in an oncological context. This is in line with the guidelines by Davies et al. (2016) that state that the 2MWT may be more feasible than the 6MWT if there is a high degree of fatigue, muscle weakness, or walking difficulties [17]. For the 10MeWT_self-test_, a more detailed video or initial face-to-face instruction with a physical therapist is recommended before the individual performs it alone at home to allow for proper standardization.

As in many populations [[22], [23], [24],^28^,^29^,^43^], the 2MWT results were highly correlated with those of the 6MWT in individuals with cancer. The two tests were very similar in terms of walking speed achieved, representing more of a comfortable rather than a maximal speed, despite the instruction to walk as far as possible. This was reflected by the mild to moderate values for dyspnea, HR and SpO_2_ at the end of the tests.

As the 2MWT and 6MWT performances were very similar, the 2MWT might be sufficient to demonstrate what the 6MWT assesses: the responses of the different systems (pulmonary, cardiovascular and neuromuscular, without differentiating between these systems) involved in submaximal exercise [39]. Furthermore, since the 6MWT does not seem to provide any additional information to the 2MWT, the shorter test might be sufficient in an oncological context.

In contrast to most previous studies (Supplementary material), only moderate correlations were found between the results of the 10MeWT and the 2MWT or 6MWT. The stronger correlation coefficients found in previous studies could relate to the populations considered. Indeed, most studies have been carried out in people with neurological pathologies whose consequences are more likely to have an impact on walking speed [47]. In people with cancer, the moderate correlation between walking speed over 10 meters and during two or six minutes observed in this study and in Eden's study of people with head and neck cancer (2018) [11] could be related to more strongly impaired performance of one test than the other. Indeed, the distances walked during the 6MWT and 2MWT in this study were lower than the normative values for individuals of the same age group [48,49], whereas the walking speeds during the 10MeWT were close to normative values [50]. These results were consistent with those from Eden's study, which reported greater deviations from normative values for the 6MWT than the 10MeWT [11]. Participants may have had more difficulty performing the endurance test because of balance problems [51], less motivation, cardiorespiratory limitations [2], central fatigue impacting motor performance [8] or muscle adaptations (atrophy of I-fibers, increased myosin fixation time in I-fibers, decreased mitochondrial density, and a shift to faster and more powerful IIa/IIx fibers) that occur with cancer [5,52].

The results of the 10MeWT showed that walking speed was not reduced in the sample, consistent with the results of Winters-Stone (2019) in people with breast cancer [12]. The moderate correlations between the results of the 10MeWT and the 6MWT and 2MWT confirm that this test, performed over 10 meters, is more a measure of an individual's general functional capacity and health status [53] than a test reflecting endurance and activity tolerance [11]. The 10MeWT gives a quick overview of mental-physical frailty (muscle weakness, mobility impairment, cognitive decline, or falls) or the risk of poor outcomes (disability in activities of daily living, hospitalization, or mortality) [54,55], rather than reflecting the level of desaturation or the responses of the cardiopulmonary system [56].

Although age had a minor influence on the 2MWT-6MWT correlation, a stronger correlation between the 10MeWT and the 6MWT and 2MWT was found in participants over 65 years old compared to younger participants. In these older individuals, reduced mobility and altered muscle function [57] may have impacted both tests and led to a stronger correlation. These hypotheses should be verified by further studies.

With the increasing use of telerehabilitation, monitoring people at home using self-tests is of interest and would allow intermediate assessments of the person's progress without needing to visit the hospital. Previously, Houchen-Wolf et al. (2020) highlighted that walking speed was the best option as a surrogate measurement of exercise capacity during remote cardiopulmonary rehabilitation assessments [34]. In our study, the good agreement between the 10MeWT_self-test_ and the 10MeWT confirmed that a walking speed test could potentially be used to assess walking capacity remotely. Further studies should confirm the possibility of using this test in the individual's own environment [34].

### 4.1 Limitations

We included outpatients who were able to walk without technical aids and who felt fit enough to carry out the three tests. However, these prerequisites may have induced a selection bias, reducing the generalizability of our results, for example to inpatients. In addition, our sample consisted mainly of individuals with lymphoma, a cancer that is not one of the four most common cancers [58], which may reduce the external validity of the study. Although the sample size was in line with recommendations [37], it was relatively small, preventing sub-group analysis, for example, by cancer type. As participants were asked to time themselves while performing the 10MeWT, this dual task may have resulted in a slight decrease in walking speed [59,60]. In addition, it required subjects to look at the markings on the ground. However, given the results obtained, this limitation does not seem to have been an issue for the participants tested.

## 5 Practical implications and conclusion

The 2MWT, the 10MeWT, and the 10MeWT_self-test_ are feasible tests for monitoring walking capacity in oncology. The strong correlation between the 2MWT and 6MWT results indicates that the 2MWT can be used as an alternative walking capacity assessment tool. The 10MeWT partially measures the same construct of walking capacity in walking-independent outpatients with cancer. The 10MeWT_self-test_ showed promising results but needs further investigations in ecological settings.

## Ethics statement

The study was reviewed and approved by the Geneva Ethics Commission (Geneva CCER – 2020-00126). All participants provided informed consent to participate in the study. All participants provided informed consent for the publication of their anonymized case details.

## Funding

This research did not receive any specific grant from funding agencies in the public, commercial, or not-for-profit sectors.

## Data availability statement

The data have not been deposited into a publicly available repository but will be made available on request.

## CRediT authorship contribution statement

**Aline Reinmann:** Writing – review & editing, Writing – original draft, Software, Project administration, Methodology, Investigation, Formal analysis, Data curation, Conceptualization. **Thibaud Koessler:** Writing – review & editing, Validation, Supervision, Project administration, Methodology, Conceptualization. **Alexandre Bodmer:** Writing – review & editing, Validation, Supervision, Project administration, Conceptualization. **Axelle Baud-Grasset:** Writing – review & editing, Methodology, Investigation. **Géraldine Mentha:** Writing – review & editing, Methodology, Investigation. **Joseph Gligorov:** Writing – review & editing, Validation, Supervision, Project administration. **Anne-Violette Bruyneel:** Writing – review & editing, Writing – original draft, Validation, Supervision, Project administration, Methodology, Investigation, Formal analysis, Conceptualization.

## Declaration of competing interest

The authors declare that they have no known competing financial interests or personal relationships that could have appeared to influence the work reported in this paper.
