# Supplementary material for: Feasibility, criterion and construct convergent validity of the 2-minute walk test and the 10-meter walk test in an oncological context
Source: Heliyon. 2023 Nov 10;9(11):e22180. doi: 10.1016/j.heliyon.2023.e22180 (PMC10692807; doi:10.1016/j.heliyon.2023.e22180)
Supplement: Multimedia component 1 [file mmc1.docx]

**Supplementary material**

**Supplementary material 1. Walking speeds and correlations between the 2MWT and the 6MWT in different populations**

| Population | Study | 6MWT | 2MWT | Correlation 2MWT-6MWT |
| --- | --- | --- | --- | --- |
|  |  | (m/s) | (m/s) | (Correlation coefficient) |
| Healthy adults (n = 330) | Bohannon et al., 2014 | 1.55 | 1.51 | *r* = 0.97 *(p < 0.01)* |
| Healthy adults (n = 31) | Swanson et al., 2019 | 1.20 | 1.25 | NS |
| Healthy adults (n = 74) | Roush et al., 2021 | Men 1.50; Women 1.46 | Men 1.51; Women 1.45 | *r* = 0.95 *(p < 0.05)* |
| Elderly (n = 16) | Connelly et al., 2009 | 0.65 | NS | *r* = 0.93 *(p-value not reported)* |
| COPD (n = 45) | Leung et al., 2006 | 1.02 | 1.09 | *r* = 0.94 *(p < 0.05)* |
| COPD (n = 26) | Gloeckl et al., 2016 | 1.1 | 1.25 | *r* = 0.81 *(p < 0.01)* |
| COPD (n = 59) | Johnston et al., 2017 | 0.92 | 1.10 | *r* = 0.87 *(p < 0.01)* |
| Stroke (n = 18) | Kosak and Smith, 2005 | NS | NS | *r* = 1.00 *(p < 0.01)* |
| MS (n = 50) | Gijbels et al., 2010  7.6MeWT standing start | 1.14 | 1.2 | *r* = 0.94 *(p-value not reported)* |
| MS (n = 42) | Bennett et al., 2017 | 0.67 | 0.73 | *r* = 0.96 *(p < 0.01)* |
| MS (n = 16 for values, 28 for correlations) | Scalzitti et al., 2018 | Disease category 1-2: 1.06  Disease category 3-4: 0.85 | Disease category 1-2: 1.17  Disease category 3-4: 0.96 | *r* = 0.95 *(p < 0.01)* |
| Myositis without assistive device (n = 67) | Alfano et al., 2014 | 1.07 | 1.12 | *r* = 0.97 *(p < 0.01)* |
| Neuro-muscular disease (n = 115) | Andersen et al., 2016 | 1.13 | 1.19 | *r* = 0.99 *(p < 0.01)* |
| Myasthenia gravis (n = 31) | Salci et al., 2019 | 1.29 | 1.42 | *r* = 0.88 *(p < 0.01)* |
| Children with Congenital Myotonic Dystrophy (n = 37) | Pucillo et al., 2017  2MWT measured during 6MWT | NS | 0.76 | *r* = 0.98 *(p < 0.01)* |
| Children with neuro-muscular disease (n = 77) | Witherspoon et al., 2019 | 1.23 | 1.24 | *r* = 0.9 *(p < 0.01)* |
| Lower extremities amputees (n = 86) | Reid et al., 2015 | NS | NS | *r* = 0.95 *(p < 0.01)* |
| Vertebral Compression Fracture (n = 10) | Goda et al., 2019 | 0.67 | 0.69 | *r* = 0.95 *(p < 0.05)* |
| Dementia or Alzheimer (n = 37) | Chan and Pin, 2019 | 0.54 | 0.52 | *r* = 0.93 *(p < 0.01)* |
| Psychosis (n = 50) | Vancamfort et al., 2019 | 1.08 based on the median | 1.07 based on the median | *r* = 0.69 *(p < 0.01)* |
| Depression (n = 50) | Vancamfort et al., 2020 | 1.1 based on the median | 1.09 based on the median | *r* = 0.94 *(p < 0.01)* |
| Alcohol use disorder (n = 50) | Vancamfort et al., 2021 | 1.33 based on the median | 1.34 based on the median | *r* = 0.91 *(p < 0.01)* |

**Supplementary material 1.** *Walking speeds and correlations between the 2MWT and the 6MWT evaluated in different populations by previous studies. COPD = Chronic Obstructive Pulmonary Disease, NS = Non-Specified, ns = non-significant. 6MWT = 6-Minute Walk Test, 2MWT = 2-Minute Walk Test.*

**Supplementary material 2. Walking speeds and correlations between the 10MeWT, 2MWT, and the 6MWT in different populations**

| Population | Study | 10MeWT | | 6MWT/2MWT | Correlation 10MeWT- | |
| --- | --- | --- | --- | --- | --- | --- |
|  |  | Comfortable (m/s) | Fast (m/s) | (m/s) | (Correlation coefficient) | |
| Cancer (head and neck) (n = 42) | Eden et al., 2018 | 1.44 | - | 6MWT: 1.46 | 6MWT | *r* = 0.68 *(p < 0.01)* |
|  |  |  |  |  | 2MWT | - |
| Healthy, older adult (n = 50) | Simonsick et al., 2000  20MeWT standing start | - | 1.64 | 6MWT: 1.26 | 6MWT | *r* = 0.65-0.73 *(p < 0.05)* |
|  |  |  |  |  | 2MWT | - |
| Healthy adults (n = 46) | Dalgas et al., 2012 | 1.70 | 2.56 | 6MWT: 1.98 | 6MWT | *r* = 0.26 comfortable speed ns,  *r* = 0.69 fast speed *(p < 0.05)* |
|  |  |  |  |  | 2MWT | - |
| Healthy adults (n = 74) | Roush et al., 2021 | Men 1.51  Women 1.42 | - | 6MWT:  Men 1.50 Women 1.46  2MWT:  Men 1.51 Women 1.45 | 6MWT | *r* = 0.59 *(p < 0.05)* |
|  |  |  |  |  | 2MWT | *r* = 0.63 *(p < 0.05)* |
| COPD (n = 49) | Andersson et al., 2011  30MeWT | 1.15 | 1.6 | NS | 6MWT | *r* = 0.73 comfortable speed *(p < 0.01)*  *r* = 0.78 fast speed *(p < 0.01)* |
|  |  |  |  |  | 2MWT | - |
| Chronic lung disease (n = 70) | DePew et al., 2013  4MeWT | 0.85 | - | 6MWT: 1.01 | 6MWT | *r* = 0.7 *(p < 0.01)* |
|  |  |  |  |  | 2MWT | - |
| Lung disease (n = 35) | Hirabayashi et al., 2020  4MeWT | 1.05 | - | NS | 6MWT | *r* = 0.57 *(p < 0.01)* |
|  |  |  |  |  | 2MWT | - |
| Cardiac disease (n = 1474) | Kamiya et al., 2018 | 1.04 | - | 6MWT: 1.06 | 6MWT | *r* = 0.80 *(p < 0.01)* |
|  |  |  |  |  | 2MWT | - |
| Stroke (n = 25) | Eng et al., 2002  4MeWT | 0.8 | - | 6MWT: 0.74 | 6MWT | *r* = 0.92 *(p < 0.01)* |
|  |  |  |  |  | 2MWT | - |
| Stroke (n = 17) | Kelly et al., 2003 | 0.71 | 1.03 | 6MWT: 0.84 | 6MWT | *r* = 0.91 comfortable speed (*p-value not reported)*  *r* = 0.89 fast speed (*p-value not reported)* |
|  |  |  |  |  | 2MWT | - |
| Stroke (n = 50) | Flansbjer et al., 2005 | 0.89-0.94 | 1.3-1.4 | 6MWT: 1.07-1.11 | 6MWT | *r* = 0.84-0.89 comfortable speed *(p < 0.01)*  *r* = 0.94-0.95 fast speed *(p < 0.01)* |
|  |  |  |  |  | 2MWT | - |
| Stroke (n = 36) | Tang et al., 2006  5MeWT | 0.84 | 1.15 | 6MWT: 0.95 | 6MWT | *r* = 0.79 comfortable speed *(p < 0.01)*  *r* = 0.82 fast speed *(p < 0.01)* |
|  |  |  |  |  | 2MWT | - |
| Stroke (n = 74) | Patterson et al., 2007  9MeWT | 0.51 | - | 6MWT: 0.6 | 6MWT | *r* = 0.88 *(p < 0.01)* |
|  |  |  |  |  | 2MWT | - |
| Stroke (n = 38) | Dalgas et al., 2012 | 0.69 | 0.92 | 6MWT: 0.81 | 6MWT | *r* = 0.91 comfortable speed  *r* = 0.94 fast speed *(p < 0.05)* |
|  |  |  |  |  | 2MWT | - |
| MS (n = 50) | Gijbels et al., 2010  7.6MeWT standing start  Correlation from Dalgas 2012 | - | 1.03 | 6MWT: 1.14 | 6MWT | *r* = 0.88 (*p-value not reported)* |
|  |  |  |  |  | 2MWT | - |
| MS (n = 48) | Dalgas et al., 2012  Standing start for MS | N/S | 1.37 | 6MWT: 1.21 | 6MWT | *r* = 0.95 fast speed *(p < 0.05)* |
|  |  |  |  |  | 2MWT | - |
| MS (n = 16 for values, 28 for correlations) | Scalzitti et al., 2018 | 1.19 | 1.55 | 6MWT: 1.06  2MWT: 1.17 | 6MWT | *r* = 0.97 comfortable speed *(p < 0.01)*  *r* = 0.93 fast speed *(p < 0.01)*  Fast vs. comfortable speed 10MeWT:  *r* = 0.96 *(p < 0.01)* |
|  |  |  |  |  | 2MWT | *r* = 0.92 comfortable speed *(p < 0.01)*  *r* = 0.94 fast speed *(p < 0.01)* |
| Neurologic impairment (n = 46) | Rossier and Wade, 2001  10MeWT measured during 2MWT  Correlation with time not speed | 0.74 | - | 2MWT: 0.7 | 6MWT | - |
|  |  |  |  |  | 2MWT | *r* = -0.61 (*p-value not reported)* |
| HTLV-1 with myelopathy (n = 26) | Adonis et al., 2016 | 0.6 based on the median | - | 6MWT: 0.5 based on the median | 6MWT | *r* = 0.93 *(p < 0.01)* |
|  |  |  |  |  | 2MWT | - |
| Parkinson (n = 346) | Duncan et al., 2017 | 1.18 | 1.64 | 6MWT: 1.2 | 6MWT | *r* = 0.75 comfortable speed *(p < 0.01)*  *r* = 0.79 fast speed *(p < 0.01)* |
|  |  |  |  |  | 2MWT | *-* |
| Spinal cord injury (n = 20) | Kim et al., 2004  Self-selected speed  4MeWT | 0.55 | - | 6MWT: 0.53 | 6MWT | *r* = 0.98 *(p < 0.01)* |
|  |  |  |  |  | 2MWT | - |
| Spinal cord injury (n = 18) | Van Hedel et al., 2007 Correlations from Dalgas 2012 | 0.79 | 1.1 | 6MWT: 1.11 | 6MWT | *r* = 0.93 comfortable speed (*p-value not reported)*  *r* = 0.93 fast speed (*p-value not reported)* |
|  |  |  |  |  | 2MWT | - |
| Spinal cord injury (n = 37) | Scivoletto et al., 2011 | 1.9 (IQR 1.3-2.8) | - | 6MWT: 0.63 (IQR 0.34-0.89) | 6MWT | - |
|  |  |  |  |  | 2MWT | - |
| Spinal cord injury (n = 33 of 95 with complete independence) | Amatachaya et al., 2014 | 0.83 | - | 6MWT: 0.63 | 6MWT | *r* = 0.83 *(p < 0.01)* |
|  |  |  |  |  | 2MWT | - |
| Spinal cord injury (n = 249) | Forrest et al., 2014 | 0.81 | - | 6MWT: 0.67 | 6MWT | *r* = 0.94 (CI95% 0.92-0.96) (*p-value not reported)* |
|  |  |  |  |  | 2MWT | - |
| Dementia or Alzheimer (n = 37) | Chan and Pin 2019  10MeWT measured during the 6MWT | 0.64 | - | 6MWT: 0.54  2MWT: 0.52 | 6MWT | *r* = 0.91 *(p < 0.01)* |
|  |  |  |  |  | 2MWT | *r* = 0.84 *(p < 0.01)* |

**Supplementary material 2.** *Walking speeds and correlations between the 10MeWT, the 2MWT and, the 6MWT evaluated in different populations by previous studies. Adapted from Dalgas et al., 2012. CI = Confidence Interval, COPD = Chronic Obstructive Pulmonary Disease, HTLV = Human T-Lymphotropic Virus, MS = Multiple Sclerosis, NS = Non-Specified, ns = non-significant. 4MeWT = 4-meter Walk Test, 5MeWT = 5-meter Walk Test, 7.6MeWT = 7.6-meter Walk Test, 9MeWT = 9-meter Walk Test, 10MeWT = 10-meter Walk Test, 20MeWT = 20-meter Walk Test, 30MeWT = 30-meter Walk Test, 6MWT = 6-Minute Walk Test, 2MWT = 2-Minute Walk Test*

**Supplementary material 3. ROC curve for 2MWT**

**Supplementary material 3.** *ROC Curve for 2MWT speed (m/s) for low mobility discrimination based on 6MWT with a threshold of 1.35 m/s (Bacquaert, 2017 from IRBMS website) provided for information. Area Under Curve = 0.93 (95%CI 0.86-1). IRBMS = Institut de Recherche du Bien-être de la Médecine et du Sport Santé,* *Institute for Welfare, Medicine and Sport Research Health, ROC = Receiver Operating Characteristic, 2MWT = 2-Minute Walk Test, 6MWT = 6-Minute Walk Test*
